# 

**Published:** 1918-09

**Authors:** 


### CONTENTS FOR THE SEPTEMBER ISSUE.

### EDITORIALS.

Boycotting the Germans.................................. 71
Nurses Needed .......................................... 72
Doctors Are Soldiers.................................... 72
Keeping Hospital Records................................ 73
Gardens About Hospitals................................. 73
Physicians Are Responding Well.......................... 74

### ORIGINAL ARTICLES.

Eczema in Childhood. By Dr. J. Spencer Davis, Dallas, Texas .......................................... 75

The Treatment of Goiter. Dr. Leigh F. Watson........... 78
Myiasis and Its Relation to Pellagra. Dr. Edward F. Wright, Greenville, Texas............................ 82

The Importance of Correlating Bedside Observation and Laboratory Methods of Diagnosis. Dr. C. E. Durham, Hico, Texas ........................................ 84

The Truth About Traumatic Hernia. Dr. Bacon Saunders, Fort Worth ........................................ 88

### ABSTRACTS AND SELECTIONS.

A Call to American Physicians, from the Red Cross....... 94
Enrollment of Physicians to Continue.................... 95
Volunteer Medical Service Corps......................... 96

Items of General Interest............................... 99

Army News ..............................................101

Relatives of Sick or Wounded Soldiers to Be Advised of Latter's Condition ....................................103

Personals ..............................................105

Helpful Hints for the Busy Doctor.......................105

Old Jokes That We Have Met..............................108

The Thirteenth Annual Meeting of the Medical Association of the Southwest.......................................104

